# Case of Hernia Cerebri, Successfully Treated by the Sponge Compress

**Published:** 1829-07

**Authors:** J. W. Heustis

**Affiliations:** Alabama


### HERNIA CEREBRI.

*Case of Hernia Cerebri, successfully treated by the Sponge Compress.* By J. W. Heustis, m.d. of Alabama.

On the 26th of March, 1828, I was called upon to visit a negro boy, about eight years of age, who had received an injury of the head. The day had been extremely windy, and many of the old dead trees in the plantations had been blown down. The subject of this article, being at work in planting corn, was struck upon the head by the falling of a limb of a tree, and knocked senseless to the earth. I found him in a state almost insensible, with frequent vomiting; pulse small, and the right eye closed. The wound was situated on the inferior and anterior angle of the left parietal bone. Upon extending the opening with a scalpel, and turning up the flaps, there appeared a depression of bone, of about the size of half a dollar, pressed down upon the brain, to the depth of about a quarter of an inch, and much splintered and shattered at the circumference. I sawed across the base of the depressed portion with Hey's convex saw, and then succeeded in elevating and removing it without having recourse to the trephine. Several small pieces of bone were driven into the substance of the brain, and were removed with the forceps. After the operation he was able to speak, which he could not do previously.

I saw him the third day after the operation: there was then no untoward symptoms. I did not remove the dressings; and, as there was some fever, I directed his bowels to be kept open with salts.

Five days after the operation I dressed the wound, which showed a disposition to become fungous: I made use of the common dressings. At the next visit I found that the fungus had protruded the opening in the skull to about the level of the integuments; I therefore removed it with the knife. From this time the growth of the fungus was rapid, so that I was under the necessity of removing it every two or three days. I at length came to the conclusion of letting it remain, and take its own course, agreeably to the advice of Mr. Abernethy. I soon, however, had reason to alter my plan; for the fungus continued to increase, and its vessels anastomosing with those of the integuments, made its growth still more rapid, producing at the same time an absorption of the scalp upon which it pressed, and a corresponding deficiency of the integuments. I now resolved to have recourse to pressure. For this purpose, having sliced away the protruding fungus to a level with the inner surface of the cranium, I applied to the wound the common dressings, lint spread with basilicon, and over this a piece of dry pressed sponge. All this was confined with a bandage passed moderately tight around the head, and under the chin, and secured by a cap.

As the patient lived several miles distant, I directed the nurse to dress the wound in the same manner, till I returned. At my next visit, two days afterwards, I found that the fungus had not increased, though, from the neglect of the nurse in suffering the bandage to become slack, but little diminution had taken place. I now increased the pressure as much as the patient could comfortably bear, and directed the attendants to continue it as long as the fungus should show a disposition to rise. Four days afterwards I visited him again, and now found that the fungus had entirely disappeared, and its place occupied by a hole in the substance of the brain as large as a hen's egg. The surface appeared healthy, being of a whitish flesh colour, and covered with a glairy exudation; the arterial pulsation had also considerably abated. I ordered all washes to be discontinued, and a simple plaster of basilicon to be applied over the hole in the cranium, guarded with the bandage and cap. I saw him no more for a week or ten days: the whole was then filled up with a healthy deposition, and the wound in a fine, healthy, and healing state. I have since learnt that this patient has perfectly recovered.

Since writing the above, I have seen, in this Journal for August 1828, p. 492, a notice of a case of Fung[illegible]us Cerebri, successfully treated by Professor Dudley on the same plan. This method of treatment by Professor Dudley had been previously mentioned to me by my young friend, Dr. Benjamin R. Hogan, of Selma; and I must do Dr. Dudley the justice to say that it was upon this information that I had recourse to pressure in the case above mentioned. I had previously deliberated with myself on the propriety of this practice, but was deterred by the unfavorable accounts respecting it recorded by surgical writers. These successful instances, however, are sufficient to authorize a further trial, and may serve to remove any existing prejudices in relation to this mode of treatment. Other means I had tried without success: the use of the knife gave no check to the morbid action, and was entirely unavailing; and caustic and strong styptic applications are in such cases inadmissible. Pressure, indeed, seems to be the only remedy that we can safely rely on with any prospect of success. Some caution may be necessary, in its employment, not to make it so great as to create pain or uneasiness, nor to continue it too long. The length of time, however, that it may be necessary, will be determined by the effect. When the disposition of the fungus to protrude has ceased, and the granulations sink below its proper level, pressure is no longer necessary. The unsightly cavity left in the brain after the removal of the fungus by pressure, might excite the apprehensions of the inexperienced practitioner; but this is a circumstance of no moment: no unfavorable symptoms are occasioned by it; nature is fully competent to the work of regeneration, and in the brain this process appears to progress with more rapidity than in any other part of the body.*

---

### HOSPITAL REPORTS,

* American Journal of Medical Science.

